# NAA20-mediated ACF1 lactylation drives neuroblastoma progression through enhancing GCLM-dependent glutathione synthesis

**DOI:** 10.1007/s10565-026-10154-7

**Published:** 2026-02-05

**Authors:** Bingqiang Han, Min Xu, Qi Wang, Jianwei Lin, Jun Chu, Yunlan Xu, Dapeng Jiang

**Affiliations:** 1 Department of Pediatric Orthopedics, Shanghai Children’s Medical Center, Shanghai Jiao Tong University School of Medicine, Shanghai, 200127 China; 2 Department of Department of Oncology (Ward 2), Shanghai Children’s Medical Center, Shanghai Jiao Tong University School of Medicine, Shanghai, 200127 China; 3 Shanghai Key Laboratory for Nucleic Acid Chemistry and Nanomedicine, Institute of Molecular Medicine, State Key Laboratory of Systems Medicine for Cancer, Shanghai Cancer Institute, Renji Hospital, School of Medicine, Shanghai Jiao Tong University, Shanghai, 200127 China; 4 Department of Pediatric Surgery, Shanghai Children’s Medical Center, Shanghai Jiao Tong University School of Medicine, Shanghai, 200127 China

**Keywords:** ACF1, NAA20, GCLM, Lactylation, Glutathione

## Abstract

Neuroblastoma (NBL) is a pediatric malignancy with poor prognosis in high-risk cases. This study explores the function of albumin conformation factor 1 (ACF1) in NBL progression and delves into the underpinning mechanism. Exome and transcriptome sequencing were applied to analyze ACF1 mutations/expression in NBL tissues versus controls. ACF1 was knocked down in NBL cell lines (KELLY, BE2C, N2a) for in vitro assays (viability, proliferation, migration, apoptosis, therapy sensitivity) or in vivo xenograft/metastasis models with radiation/cisplatin. Mechanisms were probed via RNA-sequencing, chromatin immunoprecipitation, luciferase assays, co-immunoprecipitation, and immunofluorescence assays. Expression patterns and the correlations between ACF1, GCLM, and NAA20 were detected in human NBL tissue microarrays. ACF1 mutations and elevated expression correlated with advanced tumor staging, high-risk factors, and unfavorable prognosis in NBL datasets and TMAs. ACF1 knockdown suppressed NBL cell proliferation, mobility, and *in vivo* tumor growth/metastasis, while enhancing cisplatin/radiation sensitivity and apoptosis. Mechanistically, ACF1 knockdown reduced GCLM transcription via decreased H3K27ac/H3K4me3/Myc at its promoter, elevating lipid peroxidation and lowering glutathione (GSH) levels. Lactate induced ACF1 lactylation and nuclear translocation, promoted by NAA20 interaction (enhanced by lactate). NAA20 knockdown phenocopied ACF1 effects, rescued by GCLM overexpression. NAA20 and GCLM were upregulated in NBL datasets/TMAs. This study suggests that the NAA20-mediated ACF1 lactylation drives GCLM-dependent GSH synthesis, promoting NBL cell growth and metastasis. Targeting this axis may improve therapy response.

**Graphical Abstract:**

This study uncovers a novel mechanism in which NAA20 promotes ACF1 nuclear translocation through lactylation, which in turn activates transcription of GCLM and enhances the antioxidant machinery of NBL cells, thereby promoting malignancy. As a result, the NAA20-ACF1-GCLM axis may represent a potential therapeutic target for NBL. Although our experiments confirm the direct interaction between NAA20 and ACF1, the precise lactylation sites on ACF1 remain uncharacterized. Further investigations are needed to determine whether NAA20 directly controls ACF1 lactylation or modulates the chromatin environment to achieve this effect.

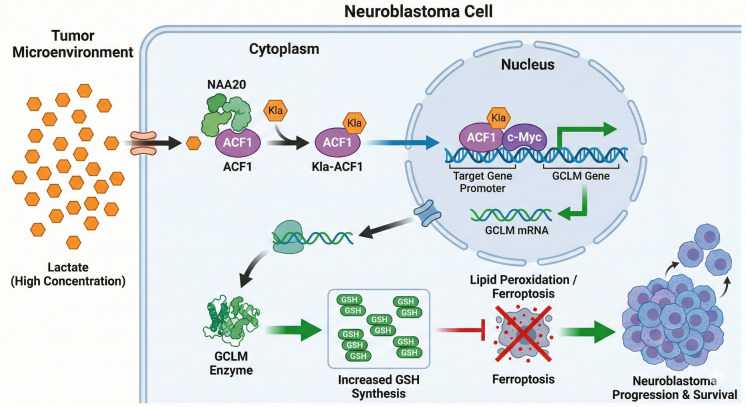

**Supplementary Information:**

The online version contains supplementary material available at 10.1007/s10565-026-10154-7.

## Introduction

Neuroblastoma (NBL) is a cancerous tumor that develops from neural crest-derived cells within the sympathetic nervous system. It represents the most frequentsolid tumor outside the brain in children, representing roughly 7%−8% of cancers in children and nearly 15% of cancer deaths in this population (Matthay et al. 2016; Smith et al. 2014; Zafar et al. 2021). NBL tumors predominantly originate in the abdominal region, particularly within the adrenal glands (Mlakar et al. 2017; Twist et al. 2019). NBL arises from neural crest-derived sympathoadrenal progenitors and exhibits a wide range of clinical behaviors. While some low-risk cases may regress spontaneously, others in high-risk groups progress aggressively (Gatta et al. 2014; Park et al. 2019; Tonini and Capasso 2020). Clinical manifestations of NBL show considerable heterogeneity, depending on variables like the patient's age at diagnosis, the anatomical site of primary tumor, and genetic markers like MYCN amplification (Abdou et al. 2012; Suzuki et al. 2018). Although combined therapeutic approaches, encompassing surgery, chemotherapy, radiation, and immunotherapy, that have boosted survival rates for high-risk patients from 15% to almost 50%, a substantial number of patients still do not respond to initial therapies (Anderson et al. 2022; Segura et al. 2022). This highlights the critical need to identify the underlying molecular pathways that fuel tumor aggressiveness and treatment resistance.

Technological advancements have provided deeper insights into the epigenome as well as epigenetic processes that regulate normal cellular activities, development, and tumorigenesis. Epigenetic mechanisms are crucial in modulating gene expression patterns that regulate cell proliferation, differentiation, and survival during both normal development and tumor progression, including in NBL (Durinck and Speleman 2018). Among various epigenetic regulators, chromatin remodeling factors have emerged as key players in tumor biology (Belk et al. 2022; Zhang and Li 2022). In particular, ISWI (Imitation SWItch)-family chromatin remodeling complexes, which include the ATP-dependent chromatin assembly and remodeling factor (ACF) complex, have been increasingly implicated in cancer progression through roles in nucleosome spacing, transcriptional regulation, DNA replication, and DNA damage repair FAch732832991# FAch732832991# FAch732832991#. One such factor, Albumin Conformation Factor 1 (ACF1), or Bromodomain Adjacent to Zinc Finger Domain 1 A (BAZ1A), is part of the ACF complex. ACF1 contributes to nucleosome positioning, DNA replication, and gene transcription (Collins et al. 2002). Together with its partner ISWI, ACF1 catalyzes nucleosome assembly in collaboration with histone chaperones (Ito et al. 1997; Ito et al. 1999). Interestingly, the ACF1 complex has been linked to the repair of double-stranded DNA breaks (Lan et al. 2010), a function that may contribute to therapy resistance in cancers exposed to DNA-damaging agents. Moreover, ACF1 has been documented as a critical transcriptional regulator promoting stemness and survival in certain tumors (Shi et al. 2025), and recent single-cell analyses in NBL cell lines have highlighted BAZ1A (ACF1) as one of the master regulators repressed by epigenetic therapies such as histone deacetylase inhibitors (Milazzo et al. 2022). Despite these insights, the precise biological functions of ACF1 in human cancers, including NBL, remain poorly understood.

One of the hallmark features of cancer is uncontrolled cell proliferation, with aberrant energy metabolism playing a key role in sustaining rapid growth (Hanahan and Weinberg 2011; Lunt and Vander Heiden 2011). Tumors typically exhibit higher glucose uptake compared to nearby healthy tissues, a process characterized by aerobic glycolysis, where glucose is transformed into lactate rather than carbon dioxide, even when oxygen is available. This process, now widely termed the Warburg effect, leads to increased lactate levels within the tumor microenvironment (TME) (Liberti and Locasale 2016; Schwartz et al. 2017). Increased lactate concentrations and the consequent acidification of the TME promote several critical processes involved in cancer progression, including angiogenesis, invasion, metastasis, and immune evasion (Chen et al. 2024).

A newly discovered post-translational modification, histone lysine lactylation (Kla), represents a unique epigenetic alteration involving the attachment of a lactyl group to lysine residues. This modification, distinct from other acylation marks, not only alters nucleosome structure but also has significant effects on chromatin dynamics and gene expression, contributing profoundly to cellular metabolic processes, inflammation, and developmental pathways (Xu et al. 2024). Emerging research suggests that chromatin remodelers are subject to diverse types of post-translational alterations that integrate metabolic signals with transcriptional regulation, influencing tumor growth and response to therapies (Liu et al. 2022; Nickerson et al. 2017; Xu et al. 2023). In light of these findings, this paper seeks to delve into the impact of ACF1 in promoting the malignant features and treatment resistance of NBL cells, with a particular focus on whether ACF1 undergoes lactylation in this context.

## Materials and methods

**Table:** 

**Key resource table**		
Reagent or Resource	Source	Identifier
**Antibodies**		
Rabbit anti-ACF1 (BAZ1A)	Abcam	Cat# ab187670
Mouse anti-Flag (Clone M2)	Sigma-Aldrich	Cat# F1804
Rabbit anti-Lactyllysine (Kla)	PTM Biolabs	Cat# PTM-1401
Mouse anti-NAA20	Santa Cruz	Cat# sc-3917
Rabbit anti-GCLM	Abcam	Cat# ab126704
Rabbit anti-GCLC	Abcam	Cat# ab190685
Rabbit anti-c-Myc	Cell Signaling Technology	Cat# 13,987
Rabbit anti-N-Myc	Cell Signaling Technology	Cat# 13,987
Rabbit anti-H3K27ac	Cell Signaling Technology	Cat# 8173
Rabbit anti-H3K4me3	Cell Signaling Technology	Cat# 9751
Mouse anti-GAPDH	Cell Signaling Technology	Cat# 97,166
Goat anti-Rabbit IgG (HRP-linked)	Cell Signaling Technology	Cat# 7074
Goat anti-Mouse IgG (HRP-linked)	Cell Signaling Technology	Cat# 7076
Alexa Fluor 488 secondary antibodies	Thermo Fisher Scientific	Cat # A-11008
**Chemicals, peptides, and recombinant proteins**		
Cisplatin	Sigma-Aldrich	Cat#232,120
Sodium Lactate (Lactate)	Sigma-Aldrich	Cat#1,614,308
Sodium Oxamate (Oxamate)	Sigma-Aldrich	Cat# O2751
Rotenone	Sigma-Aldrich	Cat# 557,368
C11-BODIPY 581/591 (Lipid Peroxidation Sensor)	Thermo Fisher Scientific	Cat# D3861
DAPI (Nuclear Stain)	Thermo Fisher Scientific	Cat# 62,248
Protein A/G Magnetic Beads	Thermo Fisher Scientific/Bio-Rad	Cat# 88,802
**Critical commercial assays**		
Cell Counting Kit-8 (CCK-8)	Dojindo	Cat# CK04
Annexin V-FITC Apoptosis Detection Kit	BD Biosciences	Cat# 556,547
Dual-Luciferase Reporter Assay System	Promega	Cat# E1910
High Capacity cDNA Reverse Transcription Kit	Applied Biosystems	Cat# 4,374,967
SYBR Green qPCR Master Mix	Applied Biosystems	Cat# A46113
ChIP-Grade Protein A/G Beads/Kit	Cell Signaling Technology	Cat# 9006
**Cell lines**		
KELLY (Human Neuroblastoma)	ECACC	92,110,411
BE(2)C (Human Neuroblastoma)	ATCC	CRL-2268
HEK293T	ATCC	CRL-3216
**Software and algorithms**		
R Project for Statistical Computing	https://www.r-project.org/	v4.0.0 or higher
GraphPad Prism	GraphPad Software	v8.0 or v9.0
ImageJ/Fiji	NIH	https://imagej.net/
Bowtie2 (ChIP-seq alignment)	Langmead and Salzberg, 2012	v2.3.5
MACS2 (Peak calling)	Zhang et al., 2008	v2.1.1
deepTools (Heatmaps/Profile plots)	Ramírez et al., 2016	v3.3.0
DiffBind (Differential binding)	Ross-Innes et al., 2012	R package

### Cells

Human NBL cell lines KELLY (CL-1084) and SK-N-BE (2)C (BE2C; CL-0441), and the HEK-293 T cell line (CL-0005) were sourced from Procell Life Science (Wuhan, China). All cells were maintained in DMEM (high glucose) containing 10% FBS and 1% antibiotics, maintained in a humidified incubator at 37℃ with 5% CO_2_ to ensure normal growth and metabolism. Cells were kept in the exponential growth phase prior to experimentation to ensure stability and reliability of results. For gene knockdown, short hairpin RNAs (shRNAs) targeting ACF1 or NAA20 were administered to cancer cell lines using lentiviral vectors, with shGFP (Green fluorescent protein) applied as control. For gene overexpression, a GCLM overexpression vector (pCMV6-GCLM) was administered utilizing Lipofectamine 2000 (Thermo Fisher Scientific). After transfection, stable clones were selected using puromycin 72 h later.

The Flag-ACF1 expression vector was loaded into HEK293T cells. After 48 h, cells were collected for further analysis. ACF1 overexpression was verified by Western blot (WB) analysis and immunofluorescence staining using an anti-Flag antibody, ensuring successful cellular expression. To test the impact of lactate on ACF1 lactylation, cells were exposed to Oxamate, a lactate dehydrogenase inhibitor. Cells were cultured with 0.1 mM Oxamate for 24 h, and ACF1 lactylation was determined by WB using an anti-lactylated lysine antibody, alongside evaluation of ACF1 nuclear localization. To further explore the role of glycolysis in ACF1 lactylation, cells were exposed to Rotenone, an inhibitor of mitochondrial respiratory chain complex I, to promote glycolysis. Cells underwent exposure to varying concentrations of Rotenone (0, 1, 2 μM) for 24 h, followed by WB examination of ACF1 lactylation and nuclear localization. Additionally, a lactate assay kit was employed to evaluate cellular lactate levels, assessing changes in glycolysis.

### Exome sequencing analysis

Tumor tissue, blood, and 3D-cultured organoid tissues from five NBL patients were subjected to exome sequencing. DNA was isolated from all samples and sequenced using the Illumina platform. DNA quality control was performed prior to sequencing to ensure integrity and purity met analysis requirements. Exome sequencing data were aligned with high quality using the Genome Analysis Toolkit. Samtools was used for data processing and filtering, followed by mutation detection with VarScan and Mutect2. To ensure high-confidence variant calling, mutations were filtered based on the following criteria: (1) a read depth > 50x, (2) a variant allele frequency (VAF) > 5% in tumor samples, and (3) a ‘PASS’ status assigned by the calling algorithm. Variants present in the matched blood controls were excluded to filter out germline polymorphisms. The ACF1 gene region was analyzed in depth to identify potential mutations or amplifications.

### Bioinformatics analysis

Transcriptome profiling data and clinical annotations for neuroblastoma were obtained from the GEO database, specifically utilizing the Kocak cohort (GSE45547, *n* = 649) profiled on the Agilent-020382 platform and the SEQC cohort (GSE62564, *n* = 498) obtained via RNA-sequencing. Data preprocessing and quality control were performed in the R statistical environment (v4.x); for microarray data (GSE45547), background correction and quantile normalization were conducted using the limma package, while RNA-seq count data (GSE62564) were normalized using the DESeq2 package after filtering low-abundance genes (CPM < 1). Differential expression analysis of ACF1 (BAZ1A) across INSS stages and risk groups was performed using limma for microarray and DESeq2 for RNA-seq data, with significance defined as an adjusted *p*-value < 0.05 and |log2 Fold Change|> 1, and prognostic value was assessed via Kaplan–Meier survival analysis using the survival package.

### RNA isolation and quantification

Cellular RNA was extracted employing the TruSeq RNA Sample Preparation Kit (Illumina) adhering to the producer’s guidelines. The RNA samples were then assessed for concentration and purity. Next, 500 ng of RNA was used for cDNA analysis utilizing the PrimeScript RT Reagent Kit (Takara, Japan). Quantitative polymerase chain reaction (qPCR) was conducted with SYBR Green Master Mix (Applied Biosystems, USA) on the ABI 7500 Real-Time PCR system. Relative gene expression was determined using the 2^–ΔΔCt^ method, with GAPDH employed as the housekeeping gene.

### RNA sequencing (RNA-seq) analysis

Total RNA was extracted from ACF1-knockdown and control KELLY cells as mentioned above, and libraries were constructed. Library quality was determined with an Agilent Bioanalyzer to ensure fragment size and concentration met high-throughput sequencing requirements. RNA samples were sequenced on the Illumina HiSeq platform, and raw data were stored in FASTQ format. Sequencing data were aligned using STAR software (v2.7) with the Ensembl-annotated genome as the reference. Aligned data were processed using samtools to ensure quality and accuracy, removing low-quality alignments. Differential expression analysis was performed using DESeq2, involving data normalization, filtering of low-expression genes, and calculation of gene expression levels and significant differences. Genes with an FDR-adjusted *p*-value < 0.05 were deemed significantly differentially expressed genes (DEGs). Gene Ontology (GO) and Kyoto Encyclopedia of Genes and Genomes (KEGG) were performed with the clusterProfiler package to elucidate ACF1-regulated cellular components, biological processes, molecular functions, and its potential role in NBL. Enrichment analysis results were visualized using ggplot2 in R.

### Lipid peroxidation assays

To measure lipid peroxidation, the C11-BODIPY 581/591 probe (Thermo Fisher) was employed. Cells were seeded in six-well plates and exposed to the indicated experimental conditions. The cells were then loaded with 10 μM C11-BODIPY 581/591 diluted in fresh medium and incubated for 30 min at 37℃ in the absence of light. Post-incubation, the cells underwent three washes with PBS, were trypsinized to detach them, and were finally resuspended in PBS. The oxidized fraction of the probe was analyzed using flow cytometry (BD Biosciences) with excitation at 488 nm and emission detection in the FITC channel.

### WB analysis

Cells or tissues were disrupted using RIPA buffer, and protein content was evaluated via the BCA assay. Uniform protein quantities (20–40 μg) were resolved by SDS-PAGE and then loaded to PVDF membrane (Millipore, USA). After blocking with 5% BSA, the membranes were probed at 4℃ with antibodies against ACF1, GCLM, NAA20, and GAPDH overnight. The next day, the membrane was incubated with an HRP-conjugated IgG for 1 h, and protein bands were developed utilizing an ECL detection reagent (Thermo Fisher). Chemiluminescent images were captured with a Tanon 5200 imaging system, and grayscale values were quantified using ImageJ software.

### Immunohistochemistry (IHC)

To assess ACF1 expression in NBL, commercial TMA samples containing tumor and adjacent tissues from 82 patients were subjected to IHC analysis. Tissue samples were paraffin-embedded and sectioned. Sections underwent dewaxing and antigen retrieval to restore antibody binding capacity. After blocking, these sections were treated with an ACF1-specific primary antibody (Abcam) at 4℃ overnight. The following day, sections were exposed to an HRP-conjugated IgG for 1 h, followed by DAB (3,3'-diaminobenzidine) staining. Sections were counterstained with hematoxylin to visualize nuclear structure.

### CCK-8 assays

KELLY and BE2C cells were plated in 96-well plates at 5,000 cells per well. After 24 h, CCK-8 reagent (Dojindo; 10 μL per well) was supplemented, followed by another 4-h incubation. Optical density was determined at 450 nm utilizing a microplate reader. Cell proliferation activity was evaluated by measuring changes in OD values.

### EdU labeling assays

KELLY and BE2C cells were plated in 96-well plates. After 48 h of incubation, 10 μM EdU solution was supplemented, and incubation continued for an additional 1 h to pulse-label DNA-replicating cells. Staining was conducted with the Click-iT EdU assay kit (Invitrogen) adhering to the supplier’s protocols. Stained cells were analyzed under a fluorescence microscope (Olympus) and quantified with Image J.

### Colony formation assay

KELLY and BE2C cells were plated in six-well plates at 500 cells per well to ensure sufficient cell numbers for independent colony formation. Cells were cultured under standard conditions for 2 weeks, with regular medium changes. Colonies were fixed for 0.1% crystal violet staining for 10 min, followed by gentle washing with water to remove unbound dye. Colonies were counted as a single colony under visual inspection.

### Drug sensitivity assays

KELLY and BE2C cells were exposed to graded cisplatin concentrations (0, 0.01, 0.1, 1, 10, and 100 μM) or exposed to varying radiation doses (0, 1, 2, 5, and 10 Gy). After an incubation period of 48 h post-treatment, cell viability was determined with CCK-8 assays as detailed above. Similarly, for apoptosis analysis, cells were harvested 48 h after treatment with 10 μM cisplatin or 5 Gy radiation. Half maximal inhibitory concentration values of cisplatin or radiation were calculated by fitting a logistic regression curve.

### Animal experiments

To validate the role of ACF1 in NBL, C57BL/6 mice (male, 6–8 weeks old) were used to establish a tumor model via subcutaneous or tail vein injection. Mouse NBL cells N2a (Procell; 1 × 10^6^ cells in 100 μL PBS) with stable artificial ACF1 knockdown were implanted into the right flank subcutaneously or via the tail vein. After 7 d, treatments were initiated, including radiation therapy (10 Gy) or cisplatin (5 mg/kg). Radiation therapy was administered weekly for three weeks using a small-animal radiation device. To prevent systemic toxicity, the radiation was delivered as localized irradiation targeting only the subcutaneous tumor on the right flank. A lead shield was employed to protect the rest of the mouse body from radiation exposure. Tumor and metastatic tissues were collected post-experiment for hematoxylin and eosin (HE) staining to analyze tumor growth, metastasis, and histological changes. Tumor volume and mouse survival were measured to evaluate survival outcomes, analyzed using the Kaplan–Meier method.

### Immunofluorescence staining

To analyze ACF1 nuclear distribution in HEK293T cells, cells were seeded in 8-well slides and transfected with the Flag-ACF1 vector. After 48 h of culture to ensure ACF1 expression, cells were fixed, permeabilized, washed, and pre-blocked with 5% BSA. Cells were exposed to an anti-Flag antibody (1:500, Invitrogen) for 1 h, followed by a 30-min incubation with an Alexa Fluor 488-conjugated IgG (1:1000, Thermo Fisher) for 30 min. Nuclei were stained with DAPI, and fluorescent images were recorded using a fluorescence microscope. The proportion of nuclear-localized Flag-ACF1-positive cells was quantified using ImageJ.

### Co-immunoprecipitation (Co-IP)

To detect physical interactions between NAA20 and ACF1, HEK293T cells were administered the Flag-ACF1 expression vector and washed. Cells were lysed in RIPA buffer, sonicated, and centrifuged to remove debris. The supernatant was treated with an anti-Flag antibody (1:500, Sigma) and Protein A/G magnetic beads (Santa Cruz) overnight to form immune complexes. After washing to remove non-specific binding, co-precipitated NAA20 protein was analyzed by WB using an anti-NAA20 antibody (1:1000, Abcam).

### Chromatin immunoprecipitation-sequencing (ChIP-seq) and ChIP-qPCR

To investigate ACF1’s role at the chromatin level, ChIP-seq was performed. Chromatin was extracted from ACF1-low-expression cells and fragmented into 200–500 bp segments using nitrogen disruption. Chromatin was incubated with anti-H3K27ac, anti-H3K4me3, and anti-Myc antibodies (1:500, Abcam) to form immune complexes, which were precipitated using Protein A/G magnetic beads. Precipitated chromatin was washed, reverse-crosslinked, and purified utilizing the phenol/chloroform method. Purified DNA was sequenced on the Illumina platform. ChIP-seq data were analyzed using Homer software to identify binding sites in the GCLM promoter region and assess their regulation of epigenetic modifications. Raw sequencing reads underwent quality control filtering and were subsequently mapped to the human reference genome (hg19) with Bowtie2. Duplicate reads originating from PCR amplification were eliminated using Picard tools. Peak detection was then carried out employing MACS2 with a q-value cutoff of 0.05. For visualization, coverage tracks were created and scaled to RPKM using the bamCoverage tool from deepTools. Heatmaps and profile plots centered at the Transcription Start Sites (TSS) were generated using computeMatrix and plotHeatmap. DiffBind, an R-based package, was used for performing differential binding analysis, comparing normalized read counts within consensus peak sets between shGFP and shACF1 groups.

Furthermore, ChIP assays were employed to analyze the interaction between ACF1 and the GCLM promoter. Briefly, the procedure began with crosslinking of KELLY and BE2C cells using 1% formaldehyde at 22–25℃ for 10 min. After lysis of the cells, chromatin was mechanically sheared into fragments of 200–500 base pairs. Each ChIP reaction contained 10 μg of chromatin and 2–4 μg of the specific antibody, with anti-ACF1 (Abcam) used for immunoprecipitation and IgG as the isotype control for the negative control. After overnight incubation with Protein A/G magnetic beads, the chromatin was eluted, reverse-crosslinked, and the DNA was purified. The enrichment of GCLM promoter sequences was assessed by qPCR.

### Luciferase reporter assays

To evaluate ACF1’s regulation of GCLM promoter transcriptional activity, a pGL4 luciferase reporter vector containing the GCLM promoter was co-transfected with the Flag-ACF1 expression vector into HEK293T cells. After 48 h, luciferase activity was determined utilizing the Luciferase Assay Reagent and analyzed via relative light units (RLU) to assess ACF1’s regulation of GCLM promoter activity. Results were analyzed using a luminometer.

### Tandem affinity purification coupled with mass spectrometry (TAP-MS)

HEK293T cells administered the Flag-ACF1 vector were lysed, sonicated, and centrifuged to remove debris. The supernatant exposed to an anti-Flag antibody (1:500, Sigma) and Protein A/G magnetic beads (Santa Cruz) to form immune complexes. After multiple washes, reverse-crosslinking, and protein degradation, purified proteins were analyzed by liquid chromatography-tandem mass spectrometry employing a mass spectrometer (Orbitrap). Data were processed using MaxQuant or PEAKS software for protein identification and quantification of ACF1 and NAA20 interaction partners.

### Immunocolocalization assays

To analyze the colocalization of NAA20 and ACF1, dual-label immunofluorescence staining was performed. KELLY or BE2C cells were plated on slides, fixed for 30 min, washed, and permeabilized. Cells were blocked with 5% BSA to reduce non-specific binding and incubated with anti-NAA20 (1:500, Abcam) and anti-ACF1 (1:500, Sigma) antibodies for 1 h. Secondary antibodies (1:1000, Thermo Fisher), were applied for 30 min. Following washing and DAPI staining, cells were observed under the fluorescence microscope, and colocalization was analyzed.

### Statistical analysis

Experimental results are exhibited as the mean ± SD. Unless otherwise specified, experiments were repeated at least six times. Statistical analysis was carried out using GraphPad Prism 9.0 (GraphPad Software). Comparisons between groups were performed using an unpaired two-tailed Student’s t-test or, where appropriate, one-way or two-way ANOVA with Tukey’s post-hoc tests. A *p*-value less than 0.05 was deemed statistically significant.

## Results

### Exome mutation and expression of ACF1 is increased in NBL

**Fig. 1:**
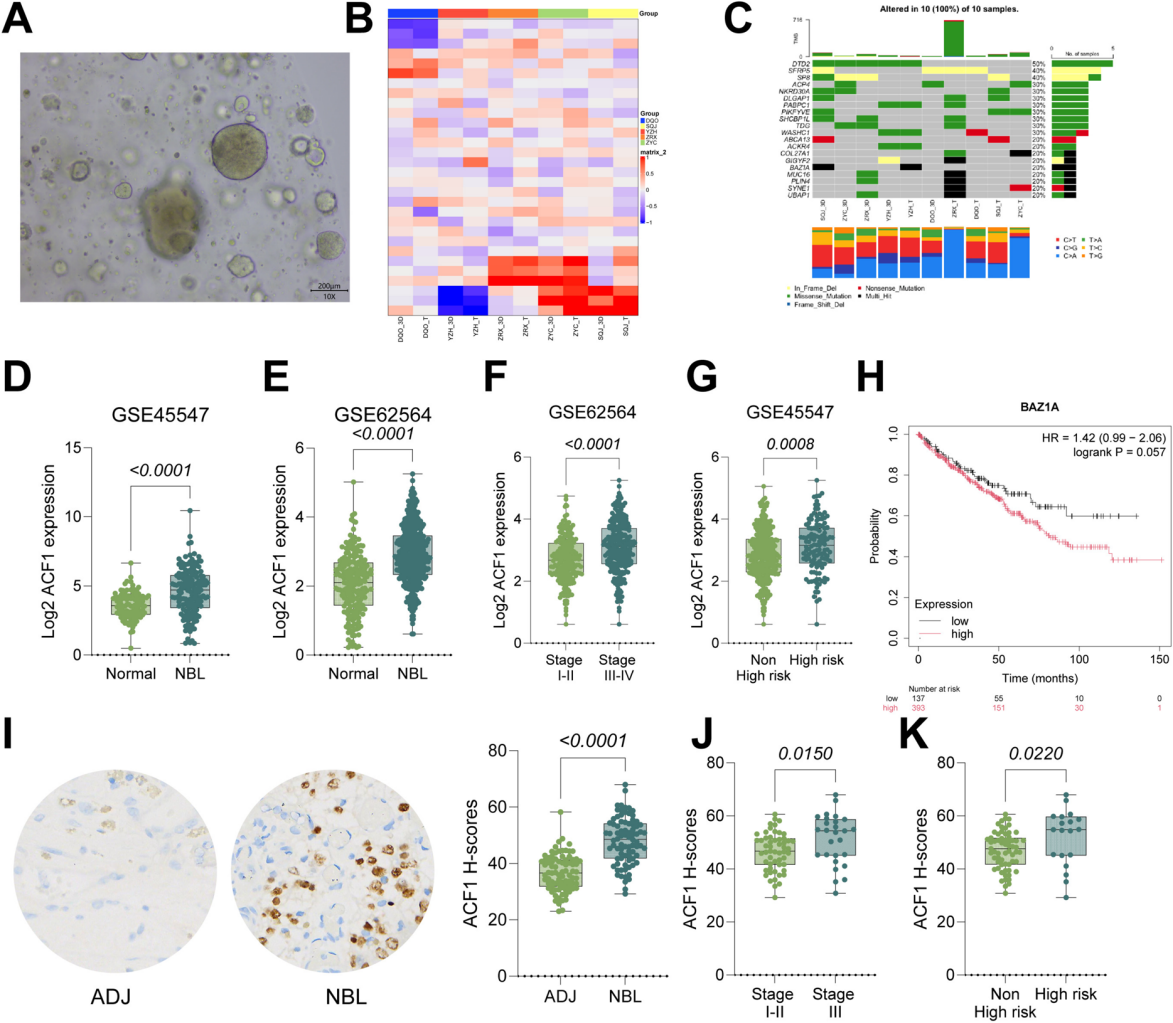
Exome mutation and expression of ACF1 is increased in NBL. **A** Representative morphology of 3D-cultured NBL organoids. **B** Heatmap showing the somatic mutation landscape of the top mutated genes identified via exome sequencing. The x-axis represents paired samples from five NBL patients, labeled by Patient ID followed by tissue type: 'T' indicates primary tumor tissue, and '3D' indicates patient-derived organoids. The y-axis lists the specific genes with identified mutations. Colors represent the mutation type/frequency as indicated in the key. Undefined abbreviations are acronyms of patients’ full names. **C** Waterfall plot summarizing the mutation frequency and type of ACF1 (BAZ1A) and other top altered genes across the five patient samples. **D-E** Analysis of ACF1 expression levels in transcriptome sequencing data from GSE45547 and GSE62564. **F-G** Correlation analysis of ACF1 expression with INSS staging and risk factors in NBL patients from the GSE62564 dataset. **H** Kaplan–Meier analysis of the correlation between ACF1 expression (labeled as BAZ1A, the gene encoding ACF1) and 5-year EFS in NBL patients. **I** IHC analysis of ACF1 staining in tumor and adjacent tissues from 82 NBL TMAs. J-K: Correlation analysis of ACF1 staining with INSS staging and risk factors in 82 NBL patients. Data are presented as dot plots and bars. Statistical significance was defined by *p* < 0.05

Exome sequencing was performed cultured organoid tissues and tumor tissues from six NBL patients, with blood samples used as controls. A recurrent amplification mutation in ACF1 (BAZ1A) was observed in NBL tumor samples (Variant Allele Frequency > 5%, Read Depth > 50x, and Mutect2 "PASS" status) (Fig. 1A-C). Detailed variant and copy number information for this cohort is provided in Table S1. Furthermore, analysis of transcriptome sequencing data from GSE45547 and GSE62564 confirmed significantly elevated ACF1 transcription levels in NBL tissues (Fig. 1D-E). In the GSE62564 dataset, ACF1 expression was markedly higher in patients with high INSS stages and those with high-risk factors compared to non-high-risk patients (Fig. 1F-G). Kaplan–Meier analysis demonstrated that patients with low ACF1 (analyzed via its gene symbol BAZ1A) expression had improved 5-year event-free survival (EFS) (Fig. 1H). Additionally, IHC analysis of TMAs showed significantly increased ACF1 staining intensity in NBL tissues compared to adjacent tissues (Fig. 1I). Align with the GSE62564 data, the clinical data from 82 patients also suggested that the ACF1 staining was higher in patients with high INSS stages versus those with low stages (Fig. 1J-K).

### ACF1 knockdown promotes growth and metastasis of NBL cells

**Fig. 2:**
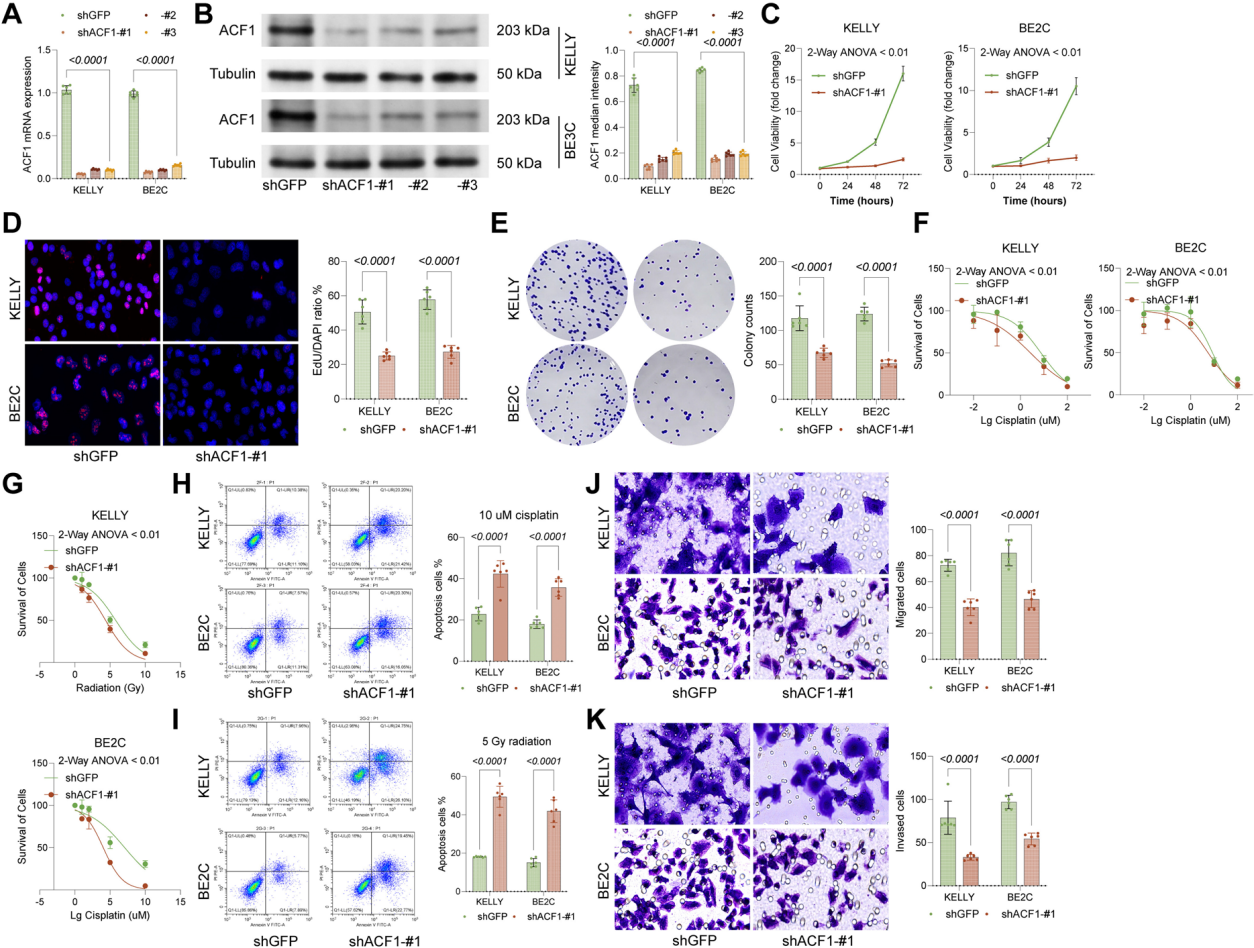
ACF1 knockdown promotes growth and metastasis of NBL cells. ACF1 shRNAs #1, #2, #3 or a control shRNA were loaded into NBL cell lines KELLY and BE3C. **A-B** ACF1 mRNA and protein levels in KELLY and BE2C cells after transfection determined using qPCR and WB analysis. The shRNA-#1 with the optimal suppressive efficacy was applied in the following experiments. **C** Viability of KELLY and BE2C cells determined using CCK-8 assays. **D** DNA replication in KELLY and BE2C cells determined using EdU assays. **E** Clonogenic capacity of KELLY and BE2C cells determined using colony formation assays. **F** Treatment of KELLY or BE2C cells with graded concentrations of cisplatin (0, 0.01, 0.1, 1, 10, 100 μM), followed by CCK-8 assays to determine IC50 values. **G** Treatment of KELLY or BE2C cells with graded radiation doses (0, 1, 2, 5, 10 Gy), followed by CCK-8 assays to determine IC50 values. **H-I** Flow cytometry detection of apoptotic cells. Note that all groups, including the shGFP control, were challenged with therapeutic stress (10 μM cisplatin or 5 Gy radiation) to evaluate chemosensitization. **J-K** Migration and invasiveness of KELLY or BE2C cells determined using Transwell assays. Six independent experiments were performed. Data are presented as dot plots and bars. Statistical significance was defined by *p* < 0.05

To further explore the influence of ACF1 in NBL, ACF1 was knocked down in NBL cell lines KELLY and BE2C (Fig. 2A-B). Compared to the shGFP control group, the ACF1 knockdown substantially suppressed cell viability (Fig. 2C). Additionally, colony formation and EdU assays showed that ACF1 knockdown markedly suppressed replication or proliferation of KELLY and BE2C cells (Fig. 2D-E). Given that chemotherapy and radiotherapy are primary NBL treatments, KELLY and BE2C cells were treated with graded cisplatin concentrations (0, 0.01, 0.1, 1, 10, 100 μM) or radiation doses (0, 1, 2, 5, 10 Gy). Knockdown of ACF1 substantially enhanced the responsiveness of NBL cells to these treatments (Fig. 2F-G). Following treatment with 10 μM cisplatin or 5 Gy radiation, the baseline apoptosis rate in control (shGFP) cells was approximately 20%. In contrast, the proportion of apoptotic cells was substantially elevated in ACF1-knockdown cells, reaching approximately 40% (Fig. 2H-I). Previous analyses indicated significantly higher ACF1 expression in high-risk patients, indicating a potential association between ACF1 expression and tumor metastasis. Indeed, Transwell assays demonstrated that the ACF1 knockdown substantially reduced migration and invasiveness of KELLY and BE2C cells (Fig. 2J-K).

### Knockdown of ACF1 restricts growth and metastasis of NBL cells in vivo

**Fig. 3:**
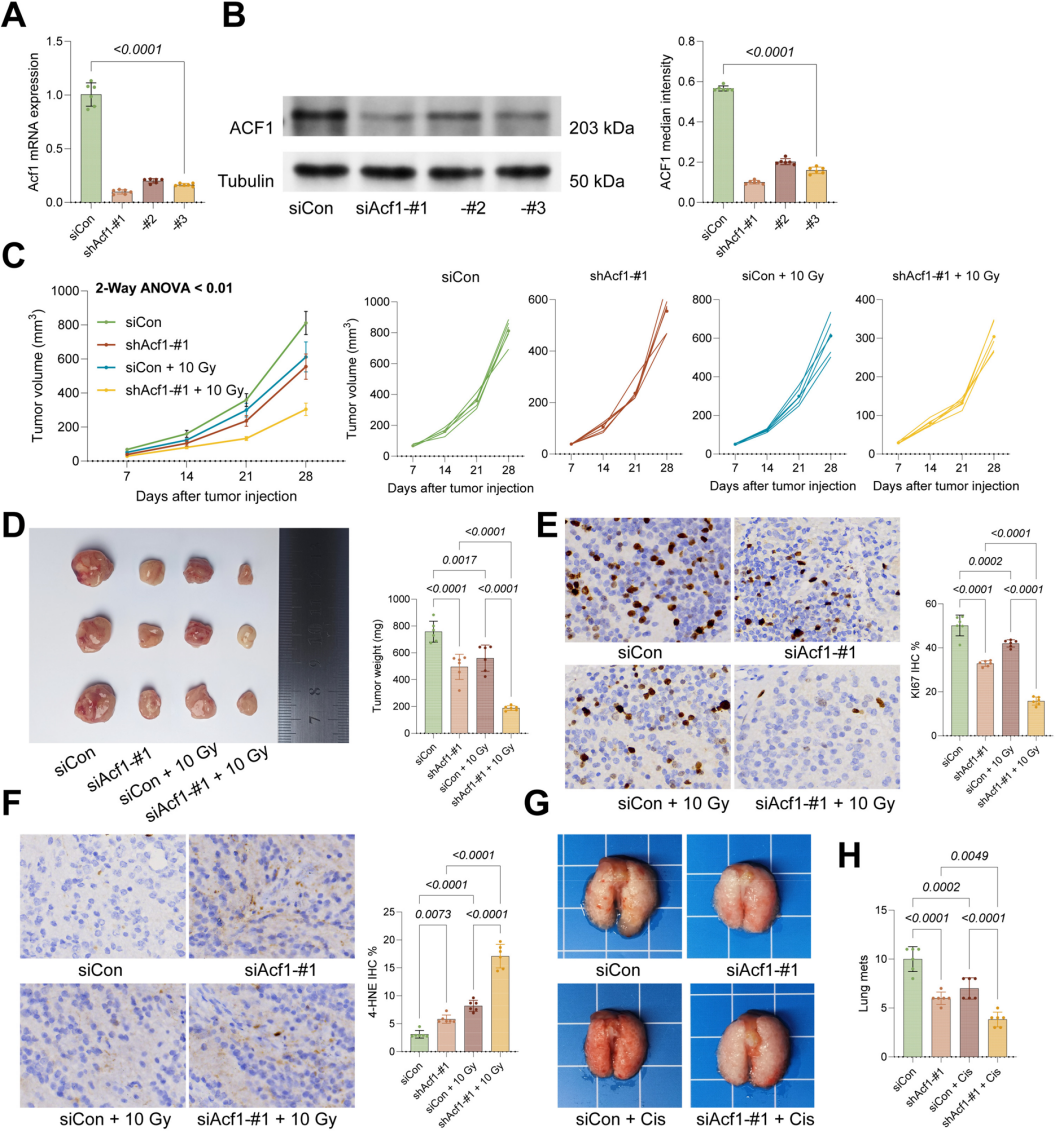
Knockdown of ACF1 restricts growth and metastasis of NBL cells in vivo. **A-B** ACF1 shRNAs #1, #2, #3 or a control shRNA were loaded into mouse NBL cells N2a, followed by qPCR and WB analyses to examine ACF1 levels in cells. N2a cells stably transfected with sh-ACF1-1# were injected into C57BL/6 mice subcutaneously, followed by radiation exposure (10 Gy) starting from day 7. **C** Volume of subcutaneous tumors in different setting. **D** Representative images and weight of the subcutaneous tumors. **E–F** Positive staining for KI67 and 4-HNE in tumor tissues determined using IHC. In another scenario, N2a cells stably transfected with sh-ACF1-1# were injected into C57BL/6 mice via the tail vein, followed by Cisplatin treatment. **G** Gross images of representative images of mouse lung tissues with metastatic nodules. **H** Number of metastatic nodules in mouse lung tissues. **I** Kaplan–Meier analysis of survival rates in another four groups of tail vein-injected metastasis model mice within 10 weeks. Each group contained six mice. Data are presented as dot plots and bars. Statistical significance was defined by *p* < 0.05

For in vivo verification, ACF1 was knocked down in mouse N2a NBL cells (Fig. 3A-B). After confirmation of the successful gene knockdown, these cells were implanted into C57BL/6 mice either subcutaneously or through the tail vein. Starting on day 7, mice with subcutaneous tumors received radiation therapy (10 Gy), while tail vein-injected mice received Cisplatin treatment. In subcutaneous models, low ACF1 expression substantially inhibited N2a cell growth in vivo and enhanced responsiveness to radiation, with radiation treatment exerting a more pronounced inhibitory effect on low-ACF1-expressing cells (Fig. 3C-D). IHC analysis revealed markedly reduced staining of the proliferation marker KI67 whereas increased staining of oxidative stress marker 4-HNE in tumors formed by low-ACF1-expressing N2a cells, particularly in radiation-treated mice (Fig. 3E-F). In tail injection models, low-ACF1-expressing N2a cells formed fewer metastatic foci in lung tissue, with further reductions observed following Cisplatin treatment (Fig. 3G-H). Kaplan–Meier analysis of survival in the metastasis model showed that ACF1 knockdown in N2a mice substantially improved survival in recipient mice, with Cisplatin treatment further enhancing survival (Fig. 3I).

### ACF1 promotes GCLM transcription and reduces lipid peroxidation in NBL cells

**Fig. 4:**
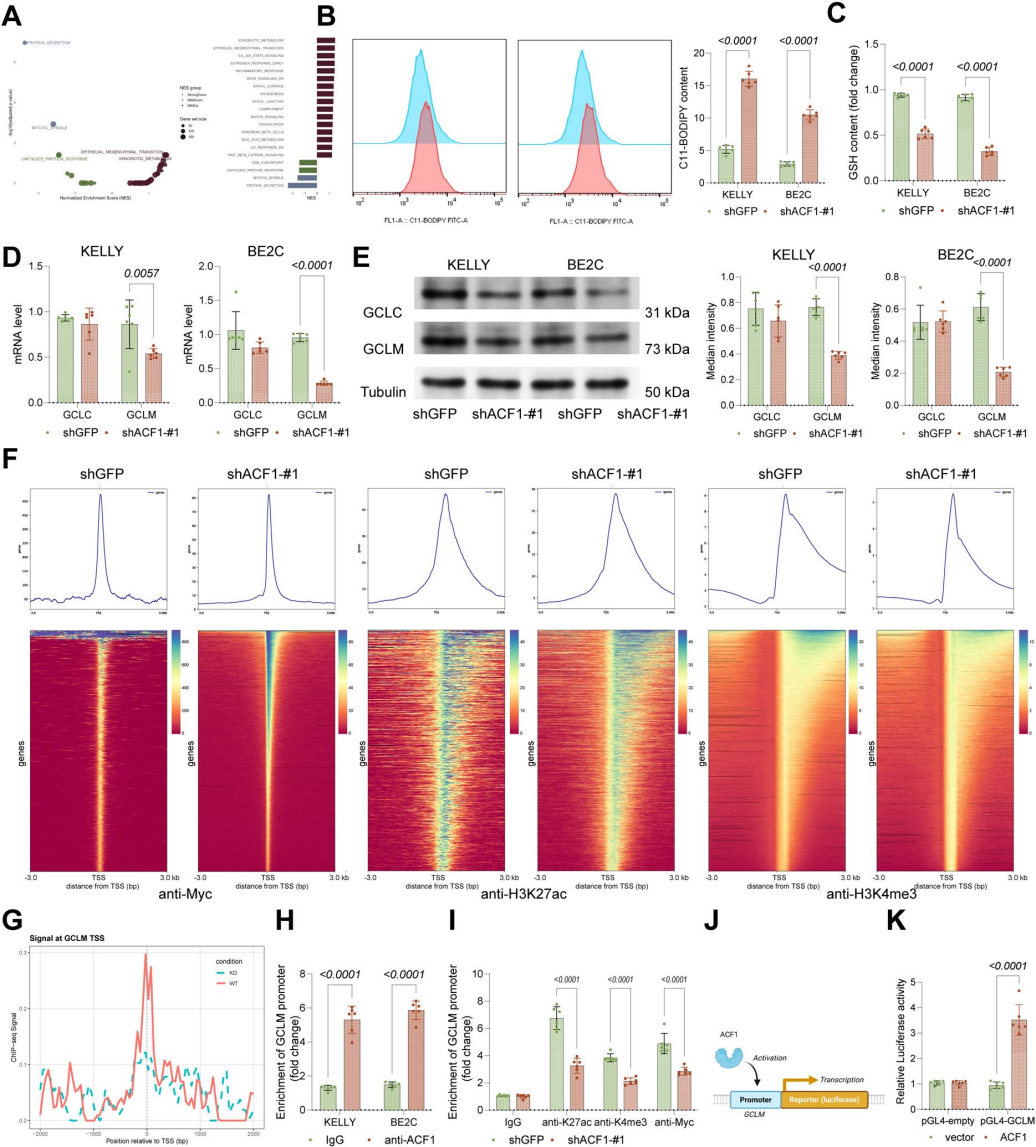
ACF1 promotes GCLM transcription and reduces lipid peroxidation in NBL cells. **A** KEGG enrichment analysis of signaling pathways enriched by DEGs following ACF1 knockdown. **B** C11-BODIPY staining in KELLY and BE2C cells with ACF1 knockdown determined using flow cytometry to assess lipid peroxidation. **C** GSH levels in KELLY and BE2C cells with ACF1 knockdown. **D-E** mRNA and protein levels of GCLC and GCLM in KELLY and BE2C cells with ACF1 knockdown determined using qPCR and WB analyses. **F-G** ChIP-seq heatmap showing binding of H3K27ac, H3K4me3, and Myc to the TSS region and the ChIP-seq data showing binding of H3K27ac, H3K4me3, and Myc to the GCLM promoter upstream region. Genes in the heatmaps are sorted in descending order of signal intensity at the TSS in the shGFP control group. The y-axes of the profile plots have been standardized to facilitate direct comparison of signal magnitude between shGFP and shACF1-#1 groups. **H** ChIP experiment using anti-ACF1, with qPCR detecting GCLM promoter content in enriched complexes. **I** ChIP experiment using anti-H3K27ac, anti-H3K4me3 and anti-Myc, with qPCR detecting GCLM promoter content in enriched complexes. **J-K** Co-transfection of a pGL4 luciferase reporter vector containing the GCLM promoter with Flag-ACF1 into HEK293T cells, followed by measurement of luciferase activity. Six independent experiments were performed. Data are presented as dot plots and bars. Statistical significance was defined by *p* < 0.05

To explore the underpinning mechanisms, RNA-seq analysis was employed to determine DEGs in ACF1-knockdown cells, followed by KEGG enrichment analysis, revealing significant enrichment in pathways such as oxidative phosphorylation, fatty acid metabolism, and glutathione metabolism, all closely related to lipid oxidation (Fig. 4A). To examine further, C11-BODIPY staining was conducted, identifying markedly increased lipid peroxidation levels in KELLY and BE2C cells with ACF1 knockdown (Fig. 4B), accompanied by a substantial reduction in intracellular GSH levels (Fig. 4C). These findings suggest that ACF1 potentially influences lipid peroxidation by regulating GSH synthesis, thereby affecting NBL growth. Among key rate-limiting enzymes in GSH synthesis, GCLM expression was significantly reduced in ACF1-knockdown cells, while GCLC expression remained unchanged (Fig. 4D-E). To determine if this regulation triggers a broader ferroptotic response, we analyzed a panel of ferroptosis-related genes (Fig. S1). We found that while other GSH synthesis enzymes were not significantly altered, the suppression of ACF1 substantially downregulated the core ferroptosis suppressor GPX4 while increasing the expression of ferroptosis markers. These data suggest that ACF1 specifically targets the rate-limiting GCLM subunit to modulate antioxidant capacity, thereby dictating the cell's susceptibility to ferroptosis (Fig. S1). Given ACF1’s role as a chromatin structure regulator, ChIP-seq was performed. In ACF1-knockdown KELLY cells, peak intensities of H3K27ac, H3K4me3, and Myc at the transcription start site (TSS) were significantly reduced (Fig. 4F), with notable decreases in H3K27ac, H3K4me3, and Myc modifications upstream of the GCLM promoter (Fig. 4G). Furthermore, ChIP experiments using anti-ACF1 antibodies confirmed the direct binding relationship between ACF1 and the GCLM promoter (Fig. 4H). Furthermore, specific ChIP-qPCR analysis verified notable decreases in H3K27ac, H3K4me3, and Myc modifications at the GCLM promoter in the presence of ACF1 knockdown (Fig. 4I), confirming reduced GCLM transcriptional activity following ACF1 knockdown. To verify ACF1’s regulation of GCLM transcriptional activity, a pGL4 luciferase reporter vector containing the GCLM promoter was co-transfected with Flag-ACF1 into HEK293T cells, demonstrating that Flag-ACF1 significantly enhanced luciferase activity (Fig. 4J-K). Furthermore, overexpression of GCLM in ACF1-low-expressing KELLY and BE2C cells (Fig. S2A-B) significantly promoted growth activity (Fig. S2C-E) and migration capacity (Fig. S2F).

### ACF1 lactylation promotes its nuclear translocation

**Fig. 5:**
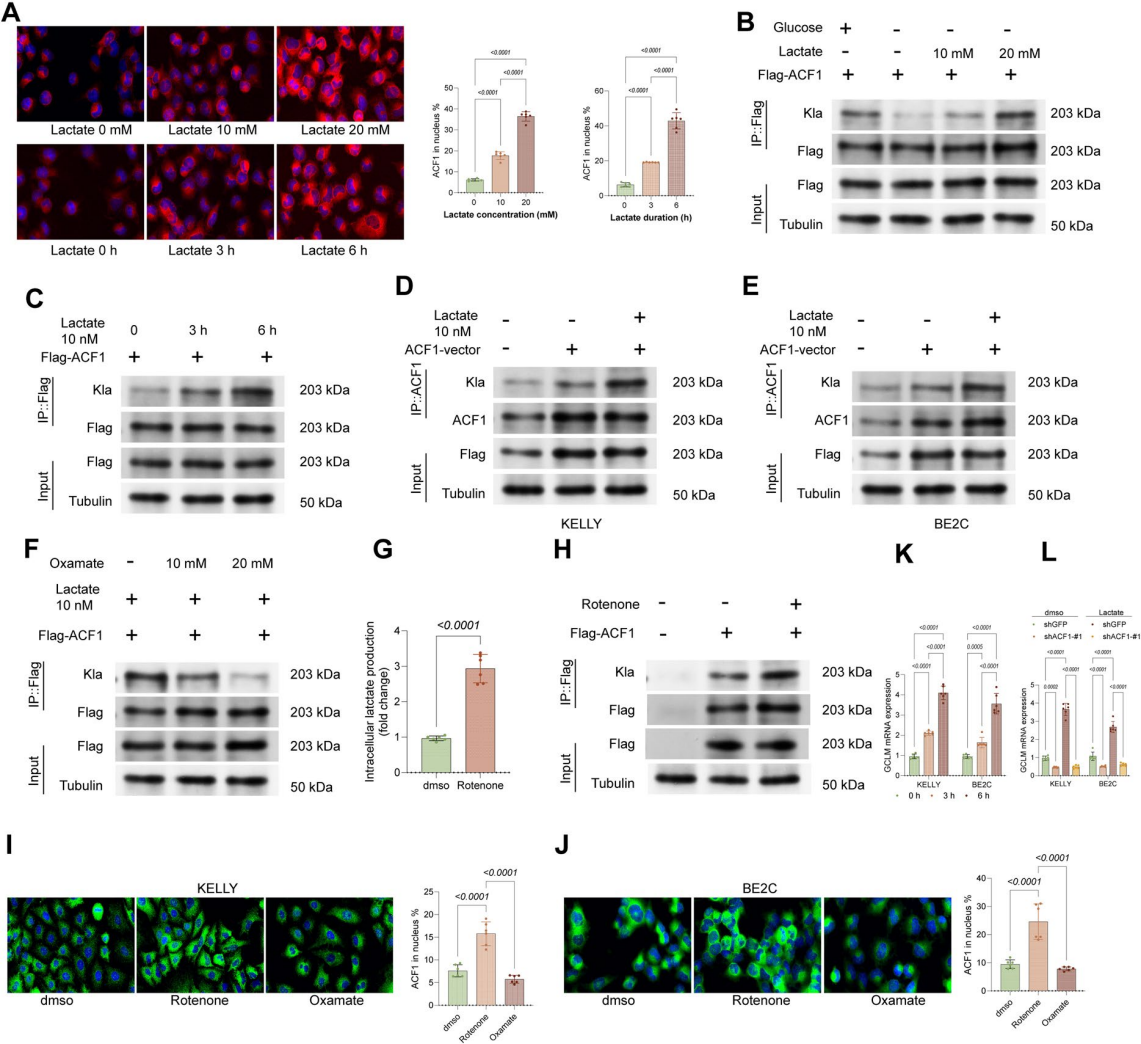
ACF1 lactylation promotes its nuclear translocation. **A** Immunofluorescence analysis of ACF1 nuclear localization. Left: Representative images showing bright-field, DAPI (blue), and Flag-ACF1 (red). Right: Quantification of the nuclear-to-cytoplasmic fluorescence intensity ratio of ACF1. **B-C** Treatment of HEK293T cells with varying lactate concentrations and durations, followed by anti-Flag IP and WB detection of Kla and Flag levels. **D-E** Exogenous ACF1 vector was administered to KELLY and BE2C cells treated with 10 mM lactate, followed by anti-ACF1 IP and WB detection of ACF1 and Kla levels. **F** Treatment of HEK293T cells with lactate dehydrogenase inhibitor Oxamate, followed by anti-Flag IP and WB detection of Kla and Flag levels. **G-H** Treatment of HEK293T cells with the mitochondrial complex I inhibitor Rotenone, followed by measurement of cellular lactate levels (G) and anti-Flag IP followed by WB detection of Kla and Flag levels. **I-J** Immunofluorescence detection of ACF1 nuclear localization in KELLY or BE2C cells treated with Oxamate or Rotenone. **K-L** qPCR analysis of GCLM mRNA levels in KELLY and BE2Ccells. (K) Cells were treated with 10 mM lactate for 6 h, showing upregulation of GCLM. (L) Cells expressing shGFP or shACF1 were treated with lactate, demonstrating that GCLM upregulation is dependent on ACF1. Six independent experiments were performed. Data are presented as dot plots and bars. Statistical significance was defined by *p* < 0.05

The TME is usually in a high lactate state. To determine whether ACF1 undergoes lactylation modification, and given that glucose is metabolized to lactate in cells, glucose starvation was applied to HEK293T cells. The Flag-ACF1 expression vector was transfected into HEK293T cells, followed by 3-h glucose starvation and subsequent supplementation with 10 mM or 20 mM lactate for 3 or 6 h. Notably, the nuclear accumulation of ACF1 was significantly increased in cells following lactate supplementation (Fig. 5A). Additionally, IP experiments with anti-Flag revealed significant lactylation modification of ACF1 after lactate treatment, with a positive correlation to lactate concentration and treatment duration (Fig. 5B-C). Additionally, we had exogenous ACF1 vector administered to KELLY and BE2C cells, followed by treatment of 10 mM lactate. Parallelly, a marked increase in ACF1 lactylation was observed in cancer cells following lactate supplementation (Fig. 5D-E). On the flip side, treatment with the lactate dehydrogenase inhibitor Oxamate significantly reduced ACF1 lactylation in Flag-expressing HEK293T cells (Fig. 5F). Furthermore, Rotenone, a mitochondrial complex I inhibitor that promotes glycolysis by blocking the electron transport chain, was administered to the HEK293T cells. This procedure substantially increased cellular lactate levels and ACF1 lactylation (Fig. 5G-H). In KELLY and BE2C cells, the Oxamate treatment significantly suppressed ACF1 nuclear localization, which was promoted by the Rotenone treatment (Fig. 5I-J). Crucially, to confirm the functional consequence of this nuclear translocation, we observed that lactate treatment significantly upregulated GCLM mRNA levels, an effect that was abrogated by ACF1 knockdown (Fig. 5K-L).

### NAA20 functions as a regulator of ACF1 lactylation

**Fig. 6:**
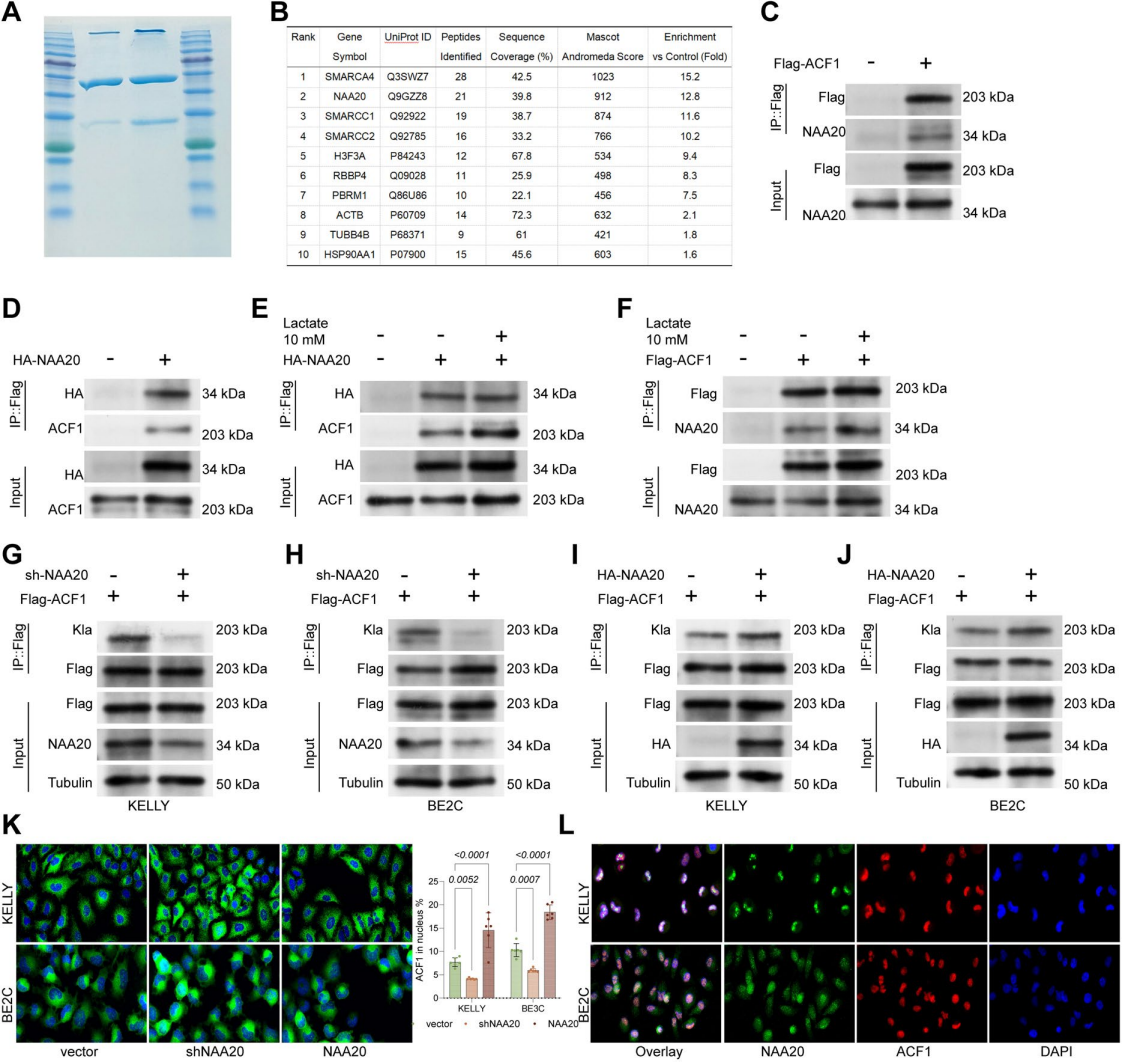
NAA20 functions as a regulator of ACF1 lactylation. **A** Coomassie blue staining of proteins binding to ACF1. **B** Identification of proteins binding to ACF1. **C** Transfection of Flag-ACF1 in HEK293T cells, followed by anti-Flag IP and WB detection of Flag and NAA20 expression. **D** Transfection of HA-NAA20 in HEK293T cells, followed by anti-HA IP and WB detection of HA and ACF1 expression. **E** HEK293T cells were transfected with HA-NAA20 for 48 h and treated with 10 mM lactate for 3 h, followed by WB detection of endogenous ACF1 in HA immunoprecipitates using anti-ACF1 antibody. **F** HEK293T cells were transfected with Flag-ACF1 for 48 h and treated with 10 mM lactate for 3 h, followed by WB detection of endogenous NAA20 in Flag immunoprecipitates using anti-NAA20 antibody. **G-H** Transfection of Flag-ACF1 and shNAA20 in KELLY or BE2C cells, followed by anti-Flag IP and WB detection of Kla levels. **I-J** Transfection of Flag-ACF1 and HA-NAA20 in KELLY or BE2C cells, followed by anti-Flag IP and WB detection of Kla levels. **K** Immunofluorescence detection of ACF1 nuclear localization in KELLY or BE2C cells upon NAA20 gain or loss. **L** Immunofluorescence colocalization assay confirming ACF1 and NAA20 interaction in KELLY or BE2C cells. Six independent experiments were performed. Data are presented as dot plots and bars. Statistical significance was defined by *p* < 0.05

To identify proteins regulating ACF1 lactylation, TAP-MS was performed on immunoprecipitates from Flag-ACF1-overexpressed HEK293T cells, identifying NAA20 as an ACF1-binding partner (Fig. 6A-B). Co-IP experiments identified the direct interaction between NAA20 and ACF1, with NAA20 detected in anti-Flag precipitates (Fig. 6C) and vice versa (Fig. 6D). Moreover, this NAA20-ACF1 binding was further enhanced upon lactate treatment (Fig. 6E-F). To further explore the regulation of NAA20 on ACF1, shRNA targeting NAA20 was transfected into Flag-ACF1-expressing KELLY or BE2C cells, which substantially reduced ACF1 lactylation (Fig. 6G-H). Conversely, transfection with an HA-NAA20 vector promoted ACF1 lactylation (Fig. 6I-J). Immunofluorescence staining also showed that knockdown of NAA20 significantly reduced ACF1 nuclear localization, while NAA20 overexpression promoted it (Fig. 6K). Dual-label immunofluorescence confirmed the colocalization of NAA20 and ACF1 in KELLY and BE2C cells (Fig. 6L).

### NAA20 regulates NBL proliferation and metastasis via ACF1-mediated GCLM regulation

**Fig. 7:**
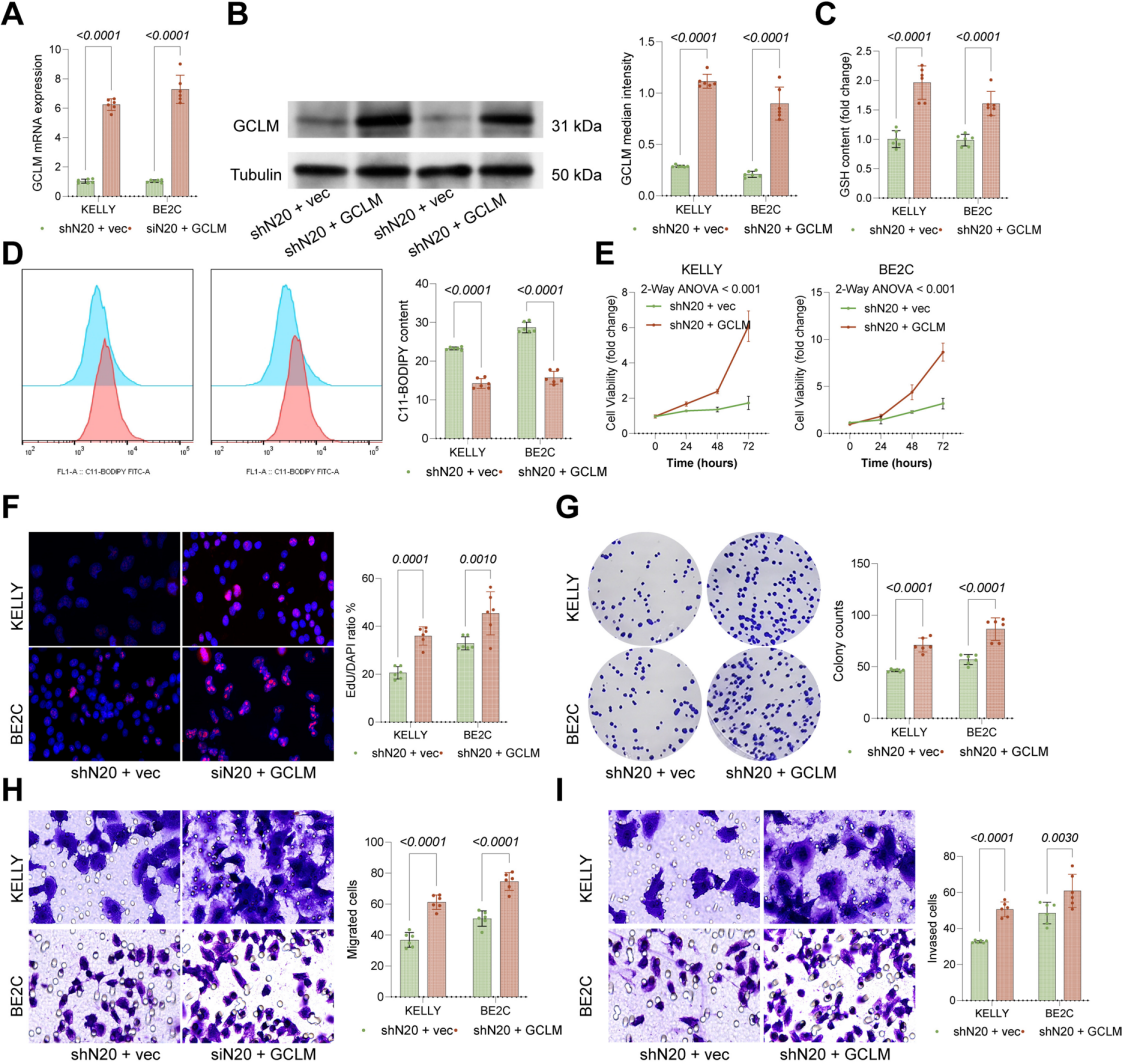
GCLM upregulation reduces lipid peroxidation and restores malignant properties in NBL cells. KELLY and BE2C cells with NAA20 knockdown were further administered the GCLM overexpression plasmid, followed by qPCR and WB analyses to verify GCLM levels. Measurement of GSH levels in KELLY and BE2C cells following GCLM restoration. **D** C11-BODIPY staining in KELLY and BE2C cells with GCLM restoration determined using flow cytometry to assess lipid peroxidation. **E** Viability of KELLY and BE2C cells determined using CCK-8 assays. **F** DNA replication in KELLY and BE2C cells determined using EdU assays. **G** Clonogenic capacity of KELLY and BE2C cells determined using colony formation assays. **H-I** Migration and invasiveness of KELLY or BE2C cells determined using Transwell assays Six independent experiments were performed. Data are presented as dot plots and bars. Statistical significance was defined by *p* < 0.05

No prior studies have linked NAA20 to tumor progression. To delve into NAA20’s impact on NBL cell growth, NAA20 knockdown was induced in KELLY and BE2C cells (Fig. S3A-B). This procedure did not significantly alter ACF1 expression but reduced ACF1 nuclear localization and GCLM expression (Fig. S3A-C). Consequently, the viability and proliferation (Fig. S3D-F), as well as the migration and invasiveness (Fig. S3G-H) of KELLY and BE2C cells were increased. Additionally, lipid peroxidation was increased while GSH production was reduced following NAA20 knockdown (Fig. S3I-J). Consistently, the GPX4 expression was decreased whereas the expression of other active ferroptosis markers was increased upon NAA20 knockdown (Fig. S1). Furthermore, the additional GCLM overexpression in NAA20-low-expressing cells (Fig. 7A-B) significantly restored GSH production (Fig. 7C) and reduced lipid peroxidation (Fig. 7D), leading to enhanced growth activity (Fig. 7E), increased EdU-positive cells, and higher colony numbers (Fig. 7F-G). Transwell assays showed significantly enhanced migration and invasion capacities in KELLY and BE2C cells following GCLM silencing (Fig. 7H-I).

### The NAA20-ACF1-GCLM axis is activated in NBL datasets and TMAs

**Fig. 8:**
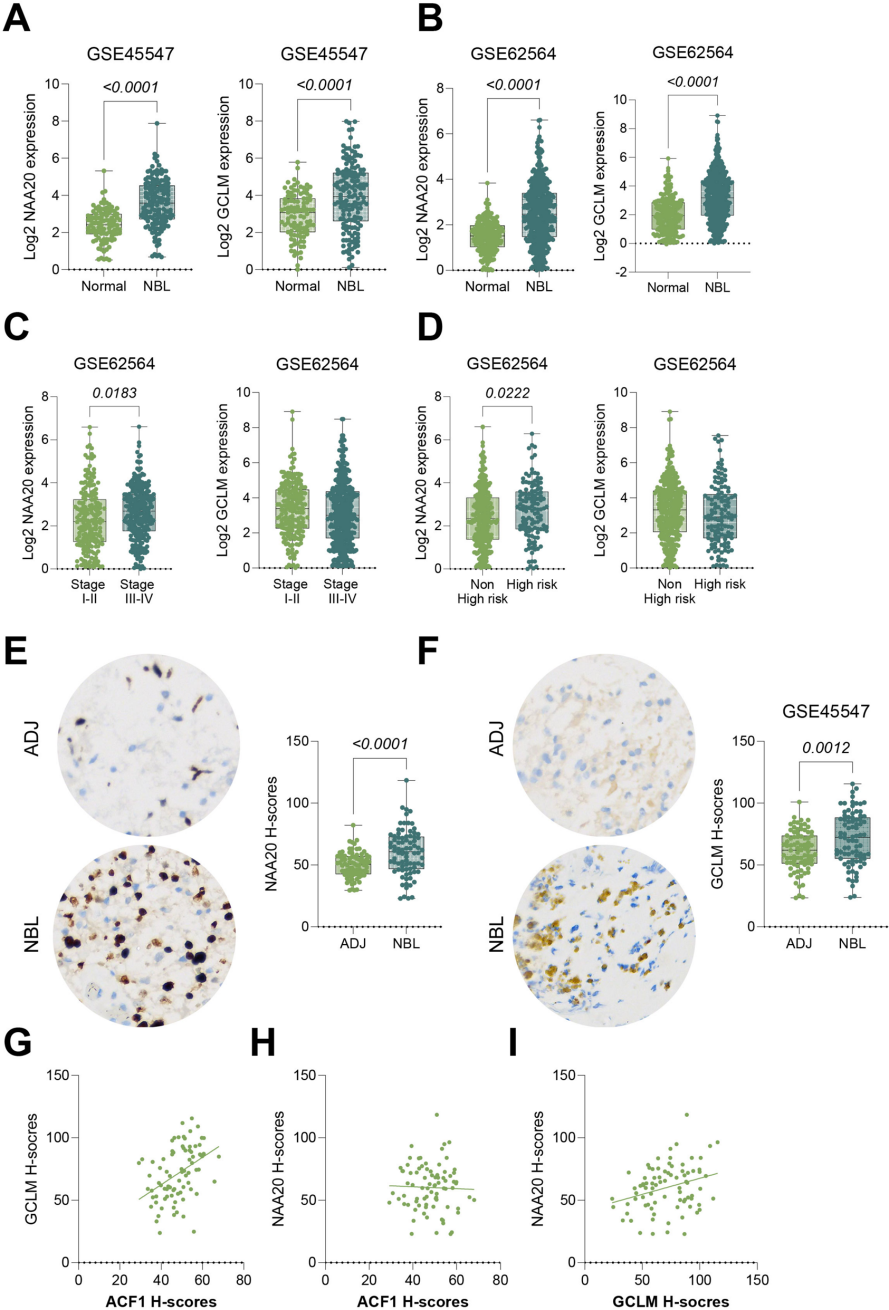
The NAA20-ACF1-GCLM axis is activated in NBL datasets and TMAs. **A-B** Analysis of NAA20 and GCLM expression in transcriptome sequencing data from GSE45547 and GSE62564 datasets. **C-D** Correlation analysis of NAA20 and GCLM levels with INSS staging and risk factors in NBL patients from the GSE62564 dataset. **E–F** IHC detection of NAA20 and GCLM staining in tumor and adjacent tissues from 82 NBL TMAs. **G-I** Pearson correlation analysis of the relationship between ACF1, NAA20, and GCLM H-scores among the 82 NBL TMAs

**Fig. 9:**
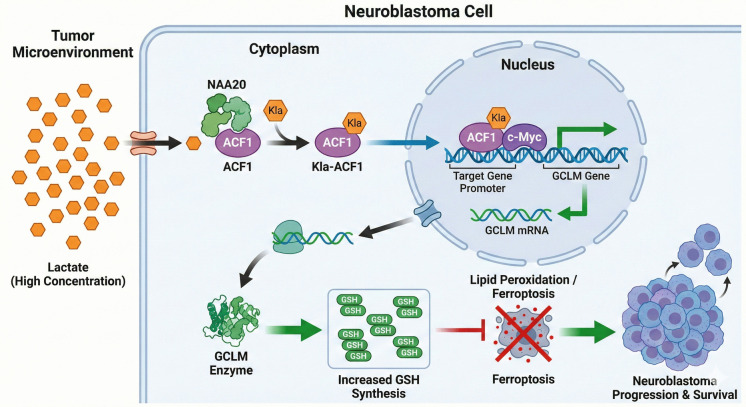
This study uncovers a novel mechanism in which NAA20 promotes ACF1 nuclear translocation through lactylation, which in turn activates transcription of GCLM and enhances the antioxidant machinery of NBL cells, thereby promoting malignancy. As a result, the NAA20-ACF1-GCLM axis may represent a potential therapeutic target for NBL. Although our experiments confirm the direct interaction between NAA20 and ACF1, the precise lactylation sites on ACF1 remain uncharacterized. Further investigations are needed to determine whether NAA20 directly controls ACF1 lactyltion or modulates the chromatin environment to achieve this effect

Further analysis of NAA20 and GCLM expression in transcriptome sequencing data from GSE45547 and GSE62564 confirmed significantly elevated expression of NAA20 and GCLM in NBL tissues (Fig. 8A-B). NAA20 expression was significantly higher in NBL patients with high-risk factors or higher INSS stages, while GCLM expression showed no significant difference (Fig. 8C-D). Additionally, IHC analysis of TMAs showed significantly increased NAA20 and GCLM staining in NBL tissues compared to the adjacent tissues (Fig. 8E-F). GCLM staining intensity was positively correlated with ACF1 staining intensity (Fig. 8G-H) but showed no significant correlation with NAA20 staining intensity (Fig. 8I), Fig. 9.

## Discussion

ACF chromatin remodelers are essential for nucleosome movement along DNA, utilizing ATP for this activity. This function is critical for DNA replication and repair, as both processes require extensive nucleosome translocation and reassembly during DNA synthesis (Erdel and Rippe 2011). The present study elucidates that the increase in ACF1 nuclear translocation following lactylation exerts oncogenic properties in NBL cells by augmenting GCLM-dependent GSH synthesis.

Large-scale genomic sequencing efforts in cancer research have recently revealed frequent mutations in chromatin regulators across various cancers (Malone and Roberts 2024). Our own exome sequencing and analysis of datasets found elevated mutations and expression of ACF1 in NBL tumors, which correlated with advanced INSS stages and reduced prognosis. Although chromatin remodelers like SWI/SNF (BAF) complexes have been studied in more detail (Baxter et al. 2023; Centore et al. 2020; Kadoch et al. 2013; Malone and Roberts 2024), the function of ACF in human cancer progression is still not fully explored. Nonetheless, a number of studies have linked ACF1 to cancer cell behavior. ACF1 has been shown to play a role in double-strand break repair (Lan et al. 2010), and Mohan et al. reported that alternative splicing of ACF1 in colorectal cancer increased chemosensitivity by disrupting the DNA damage response (Mohan et al. 2024). Furthermore, the loss of ACF1 resulted in reduced tumorigenic potential, decreased cell viability, and increased DNA damage, senescence, and apoptosis in colorectal cancer cells (Mohan et al.^2024^). Koyauchi et al*.* found that ACF1 interacts with methyltransferase MLL1 to facilitate nucleotide excision repair at ultraviolet-induced damage sites (Koyauchi et al. 2022). In hepatocellular carcinoma, ACF1 has been implicated in promoting tumor progression through integrated transcriptomic and proteomic alterations (Liu et al. 2024). Furthermore, a recent work by Shi et al. revealed that ACF1, stabilized by ubiquitin-specific peptidase 10, interacts with SRY-box transcription factor 2 to activate transcription machinery, driving cancer stem cell-related gene expression (Shi et al. 2025). Align with these insights, our findings demonstrated that knockdown of ACF1 suppressed NBL cell proliferation, mobility, and in vivo tumor growth/metastasis, while enhancing cisplatin/radiation sensitivity and apoptosis. These observations confirmed an oncogenic role of ACF1 in NBL.

Additional experiments revealed increased lipid peroxidation and decreased GSH levels in NBL cells following ACF1 knockdown. Lipid peroxidation occurs when reactive oxygen species (ROS) damage polyunsaturated fatty acids in cellular membranes, generating lipid hydroperoxides and reactive aldehydes that compromise membrane integrity and cellular function (Yang et al. 2016). This process is closely linked to programmed cell death like apoptosis (Wang et al. 2023a) and ferroptosis (Cao and Dixon 2016). In contrast, GSH is vital for preserving cellular redox homeostasis, detoxifying ROS and reactive nitrogen species, and safeguarding cells against oxidative damage and death (Ali et al. 2020; Bansal and Simon 2018; Niu et al. 2021). Targeting GSH and its antioxidant pathways has been proposed as a promising therapeutic option for NBL, especially in MYCN-amplified cases (Alborzinia et al. 2022; Floros et al. 2021). Additionally, we identified that the ACF1 loss substantially restricted GPX4 while increasing the expression of ferroptosis markers. Among the two subunits of glutamate-cysteine ligase (GCL), the key enzyme in GSH synthesis, only GCLM was significantly reduced following ACF1 knockdown in NBL cells. These data suggest that ACF1 specifically targets the rate-limiting GCLM subunit to modulate antioxidant capacity, thereby dictating the cell's susceptibility to lipid peroxidation and cell death.

Additionally, modifications indicative of active transcription, such as H3K27ac, H3K4me3, and Myc (Bartosovic et al. 2021; Beacon et al. 2021; Patange et al. 2022), were decreased in ACF1-deficient NBL cells, suggesting that ACF1 knockdown diminishes malignant characteristics and increases therapy sensitivity by inhibiting GCLM-dependent GSH synthesis. This hypothesis was partially confirmed when overexpression of GCLM in ACF1-knockdown KELLY and BE2C cells restored their malignant properties. Lactate, once thought to be merely a waste product of anaerobic metabolism, has gained attention as an important signaling molecule (Brooks 2020) and is now recognized as a key regulator of cellular reprogramming via lactylation (Lv et al. 2023). Like other epigenetic modifications, lactylation modifies proteins, including chromatin regulators, enabling the integration of metabolic signals, such as elevated lactate from glycolysis, into epigenetic control mechanisms (Wang et al. 2023b). Lactylation can influence protein behavior by either promoting nuclear retention/import or driving export, depending on the specific protein and modification site (Fan et al. 2025; Li et al. 2024; Wang et al. 2024). Additionally, we observed an increase in total ACF1 protein levels following lactate treatment. We hypothesize that lactylation at lysine residues may compete with ubiquitination sites, thereby inhibiting proteasomal degradation and stabilizing the ACF1 protein, a mechanism analogous to that observed for other stress-responsive chromatin factors.

Despite significant progress in understanding lactylation, the primary enzymes responsible for catalyzing lysine lactylation remain poorly understood. Emerging evidence in the field of acyl-modifications demonstrates that many lysine acyltransferases, originally identified as acetyltransferases, exhibit promiscuous activity and can catalyze other acylations (like lactylation, crotonylation, and succinylation) depending on the availability and intracellular ratio of acyl-CoA donors. Classic examples include p300/CBP and KAT2A (GCN5), and HBO1, which transfer lactyl groups from lactyl-CoA to lysine residues on histones and non-histone proteins, particularly under high-lactate conditions (Niu et al. 2024; Zhang et al. 2019; Zhu et al. 2025). In our study, we found that NAA20, a key component of the NatB N-terminal acetyltransferase complex, directly interacts with ACF1, promoting its lactylation and subsequent nuclear translocation.

NAA20 is primarily recognized for its function in acetylating the N-terminal methionine of approximately 20% of proteins in the human proteome (D'Onofrio et al. 2023). Interestingly, NAA20 has also been shown to influence metabolism by acetylating liver kinase B1 and inhibiting the AMP-activated protein kinase (AMPK) pathway, which accelerates hepatocellular cancer progression (Jung et al. 2020). AMPK typically suppresses glycolysis (Ghirotto et al. 2019; Huang et al. 2020), and its inhibition by NAA20 likely promotes glycolytic flux, contributing to elevated lactate production and increased availability of lactyl-CoA. This metabolic link provides a plausible basis for NAA20's non-canonical role in promoting ACF1 lactylation, potentially through acyl-CoA substrate promiscuity analogous to that observed in other acyltransferases. Furthermore, NAA20 has been implicated in driving triple-negative breast cancer through its regulation of Rab5A-mediated activation of EGFR signaling (Qiao et al. 2023). In this study, knockdown of NAA20 replicated the effects of ACF1 knockdown, suppressing malignant features of NBL cells, and these effects were reversed by GCLM overexpression. Our findings thus uncover a novel metabolic-epigenetic axis in neuroblastoma, where NAA20 bridges glycolysis-derived lactate to ACF1 modification and glutathione synthesis, warranting further investigation into its lactyltransferase-like activity *in vitro*.

Further analyses of GEO datasets and TMAs showed that NAA20 expression was markedly higher in NBL patients with high-risk factors or higher INSS stages, while GCLM expression showed no significant difference. This suggests that elevated GCLM expression may represent an adaptive response to metabolic stress or changes in tumor cells, supporting survival, antioxidant defense, and redox balance, rather than directly correlating with tumor malignancy or stage.

However, several limitations should be acknowledged. First, MYCN amplification is the primary driver in NBL biology. However, this study primarily investigates the enrichment of Myc (c-Myc), along with other active transcription markers H3K27ac, H3K4me3, near the promoter region of downstream gene (GCLM) upon ACF1 modulation. Given that c-Myc and N-Myc share high homology and bind to similar E-box motifs (Kress et al. 2015; Murphy et al. 2009), it is highly plausible that ACF1 also acts as a chromatin remodeler for N-Myc, thereby facilitating the transcriptional elongation of oncogenic targets beyond the metabolic genes identified here. Future studies are warranted to biochemically characterize the physical interaction between ACF1 and N-Myc and to dissect their combined contribution to NBL aggressiveness. Additionally, our animal studies primarily utilized the murine N2a syngeneic model. While this allowed for the assessment of tumor progression in an immunocompetent context, future studies utilizing patient-derived xenografts (PDX) or human neuroblastoma cell line xenografts are necessary to fully confirm the translational potential of targeting the NAA20-ACF1 axis in human tissues. Furthermore, while we demonstrated that ACF1 knockdown sensitizes tumors to localized radiation (subcutaneous model) and systemic cisplatin (metastasis model), combinatorial regimens exploring synergistic effects in PDX models will be crucial for clinical translation.

## Conclusion

In conclusion, this paper uncovers an innovative mechanism where NAA20 promotes ACF1 nuclear translocation through lactylation, which in turn activates transcription of GCLM and enhances the antioxidant machinery of NBL cells, thereby promoting malignancy. As a result, the NAA20-ACF1-GCLM axis may represent a potential therapeutic target for NBL. Although our experiments confirm the direct interaction between NAA20 and ACF1, the precise lactylation sites on ACF1 remain uncharacterized. Further investigations are needed to determine whether NAA20 directly controls ACF1 lactylation or modulates the chromatin environment to achieve this effect.

## Supplementary Information

Below is the link to the electronic supplementary material.

Supplementary file1 (PDF 2445 KB)

Supplementary file2 (DOCX 13 KB)

## Data Availability

The data used to support the findings of this study are available from the corresponding author upon request.

## Abbreviations

NBL: Neuroblastoma
ACF1: Albumin conformation factor 1
BAZ1A: Zinc finger domain 1A
EFS: Event-free survival
INSS: International neuroblastoma staging system
