# Supplementary material for: NAA20-mediated ACF1 lactylation drives neuroblastoma progression through enhancing GCLM-dependent glutathione synthesis
Source: Cell Biol Toxicol. 2026 Feb 5;42(1):36. doi: 10.1007/s10565-026-10154-7 (PMC12906602; doi:10.1007/s10565-026-10154-7)
Supplement: Supplementary file 1 — Supplementary file1 (PDF 2445 KB) [file 10565_2026_10154_MOESM1_ESM.pdf]

Fig. S1.

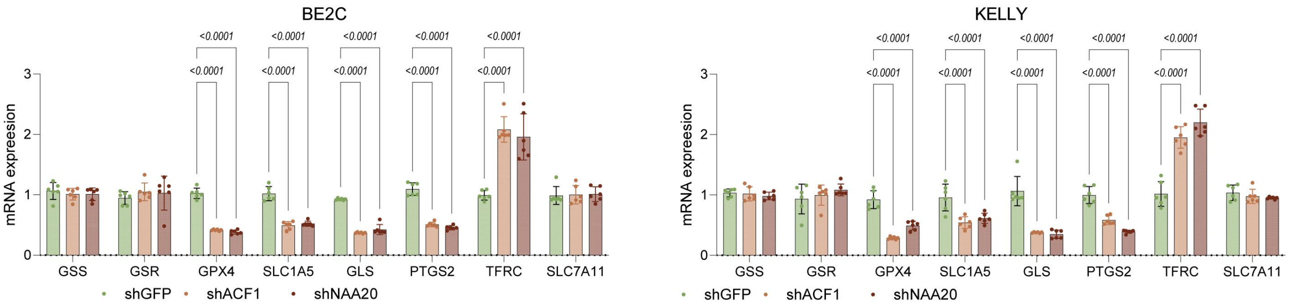

Fig. S1. Broad validation of metabolic and ferroptosis-related genes. qPCR analysis of a panel of 8 key genes involved in GSH synthesis and ferroptosis in BE2C and KELLY cells expressing shGFP vs. shNAA20 or shACF1. Six independent experiments were performed. Data are presented as dot plots and bars. Statistical significance was defined by $p < 0.05$.

Fig. S2

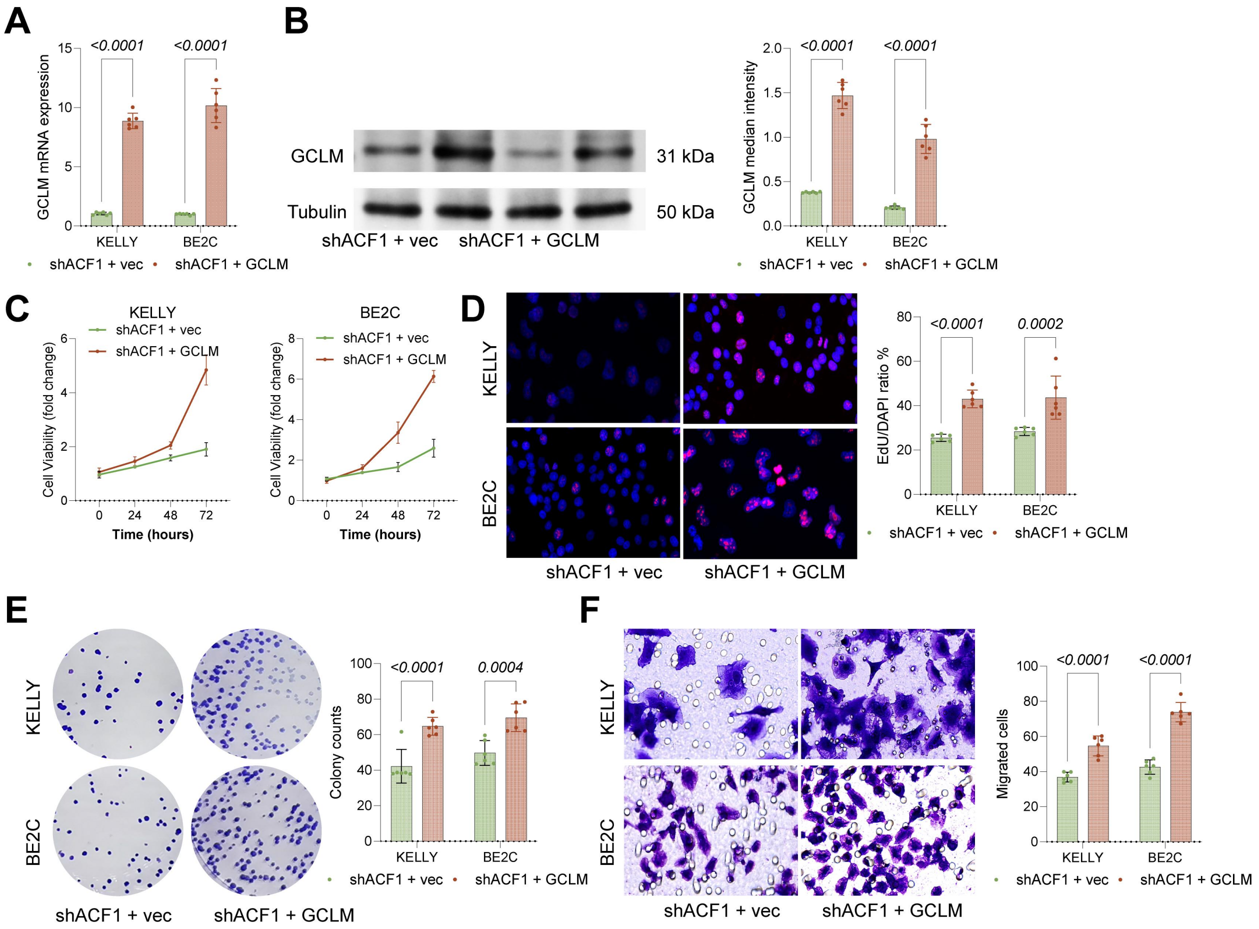

Fig. S2. GCLM overexpression restores viability and migration in ACF1-low-expressing NBL cells. KELLY and BE2C cells with ACF1 knockdown were further administered the GCLM overexpression plasmid, followed by qPCR and WB analyses to verify GCLM levels. C: Viability of KELLY and BE2C cells determined using CCK-8 assays. D: DNA replication in KELLY and BE2C cells determined using EdU assays. E: Clonogenic capacity of KELLY and BE2C cells determined using colony formation assays. F: Migration of KELLY or BE2C cells determined using Transwell assays. Six independent experiments were performed. Data are presented as dot plots and bars. Statistical significance was defined by $p < 0.05$.

Fig. S3

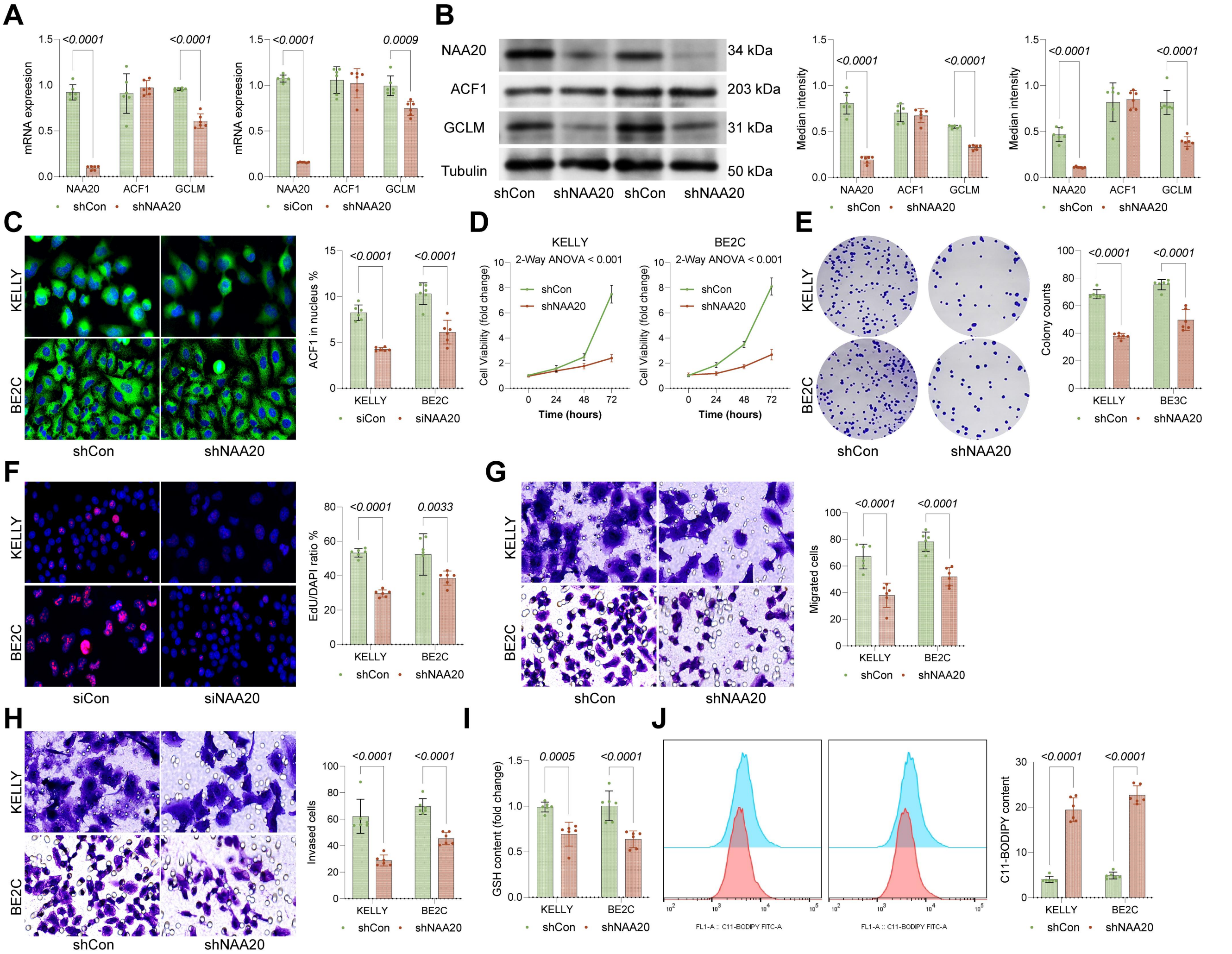

Fig. S3. Knockdown of NAA20 restricts cell growth and mobility of NBL cells by blocking the ACF1-GCLM axis A-B: Transfection of shRNA targeting NAA20 in KELLY or BE2Ccells, followed by qPCR and WB detection of NAA20, ACF1, and GCLM levels. C: Immunofluorescence detection of ACF1 nuclear localization in KELLY or BE2C cells following NAA20 knockdown. D: Viability of KELLY and BE2C cells following NAA20 knockdown determined using CCK-8 assays. E: Clonogenic capacity of KELLY and BE2C cells with NAA20 knockdown determined using colony formation assays. F: DNA replication in KELLY and BE2C cells following NAA20 knockdown determined using EdU assays. G-H: Migration and invasiveness of KELLY or BE2C cells with NAA20 knockdown determined using Transwell assays. I: Measurement of GSH levels in KELLY and BE2C

cells following NAA20 knockdown. J: C11-BODIPY staining in KELLY and BE2C cells with NAA20 knockdown determined using flow cytometry to assess lipid peroxidation. Six independent experiments were performed. Data are presented as dot plots and bars. Statistical significance was defined by $p < 0.05$.
