# Supplementary material for: NAA20-mediated ACF1 lactylation drives neuroblastoma progression through enhancing GCLM-dependent glutathione synthesis
Source: Cell Biol Toxicol. 2026 Feb 5;42(1):36. doi: 10.1007/s10565-026-10154-7 (PMC12906602; doi:10.1007/s10565-026-10154-7)
Supplement: Supplementary file 2 — Supplementary file2 (DOCX 13 KB) [file 10565_2026_10154_MOESM2_ESM.docx]

**Table S1.** Clinical characteristics and ACF1 (BAZ1A) genomic status of the pilot neuroblastoma patient cohort (n=6) analyzed by Whole Exome Sequencing (WES).

| **Patient ID** | **Age at Diagnosis (Months)** | **INSS Stage** | **MYCN Status** | **Clinical Risk Group** | **ACF1 (BAZ1A) Copy Number Statusᵃ** | **Other Notable SNVs/Indelsᵇ** |
| --- | --- | --- | --- | --- | --- | --- |
| **NBL-PT01** | 36 | 4 | Amplified | High Risk | Amplification | *ALK* F1174L |
| **NBL-PT02** | 28 | 4 | Amplified | High Risk | Amplification | None detected |
| **NBL-PT03** | 42 | 4 | Non-amplified | High Risk | Amplification | *ATRX* deletion |
| **NBL-PT04** | 18 | 2 | Non-amplified | Low Risk | Wild-type (Diploid) | None detected |
| **NBL-PT05** | 8 | 1 | Non-amplified | Low Risk | Wild-type (Diploid) | None detected |
| **NBL-PT06** | 12 | 3 | Non-amplified | Intermediate Risk | Wild-type (Diploid) | None detected |

Note: Copy Number Status: Determined by WES bioinformatics analysis pipeline. "Amplification" indicates significant focal copy number gain (e.g., estimated copy number > 4). "Wild-type (Diploid)" indicates normal copy number (CN=2). ᵇ SNVs/Indels: Single Nucleotide Variants or small Insertions/Deletions detected in known neuroblastoma driver genes. No recurrent somatic point mutations were identified in ACF1 (BAZ1A) in this cohort. INSS: International Neuroblastoma Staging System.
